# 1-Methyl-5-phen­oxy-3-trifluoro­methyl-1*H*-pyrazole-4-carbaldehyde oxime

**DOI:** 10.1107/S1600536811007422

**Published:** 2011-03-05

**Authors:** Hong Dai, Wen-Ke Miao, Shan-Shan Wu, Xue Qin, Jian-Xin Fang

**Affiliations:** a State Key Laboratory and Institute of Elemento-Organic Chemistry, Nankai University, Tianjin 300071, People’s Republic of China; b College of Chemistry and Chemical Engineering, Nantong University, Nantong 226019, People’s Republic of China

## Abstract

In the title compound, C_12_H_10_F_3_N_3_O_2_, the dihedral angle between the phenyl and pyrazole rings is 96.6 (3)°. In the crystal, pairs of O—H⋯N hydrogen bonds link the mol­ecules, forming inversion dimers. Weak inter­molecular C—H⋯F hydrogen bonds are also observed.

## Related literature

For the biological activity of pyrazole-4-carbaldehyde oxime ether derivatives, see: Hamaguchi *et al.* (1995 ▶); Motoba *et al.* (1992 ▶).
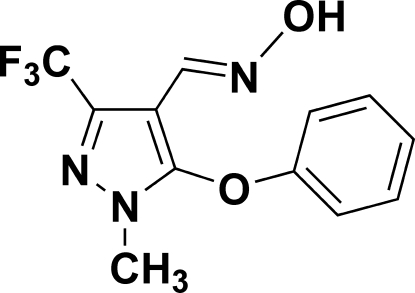


## Experimental

### 

#### Crystal data

C_12_H_10_F_3_N_3_O_2_
*M* *_r_* = 285.23
Monoclinic, 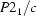

*a* = 7.5221 (15) Å
*b* = 18.282 (4) Å
*c* = 9.1002 (18) Å
β = 90.58 (3)°
*V* = 1251.4 (4) Å^3^
*Z* = 4
Mo *K*α radiation
μ = 0.14 mm^−1^
*T* = 113 K
0.24 × 0.16 × 0.14 mm

#### Data collection

Bruker SMART 1000 CCD diffractometer
Absorption correction: multi-scan (*SADABS*; Sheldrick, 1996) ▶ *T*_min_ = 0.968, *T*_max_ = 0.981
7050 measured reflections
2185 independent reflections
1910 reflections with *I* > 2σ(*I*)
*R*_int_ = 0.030

#### Refinement

*R*[*F* ^2^ > 2σ(*F* ^2^)] = 0.037
*wR*(*F* ^2^) = 0.101
*S* = 1.06
2185 reflections
183 parameters
H-atom parameters constrained
Δρ_max_ = 0.25 e Å^−3^
Δρ_min_ = −0.24 e Å^−3^

### 

Data collection: *SMART* (Bruker, 1998 ▶); cell refinement: *SAINT* (Bruker, 1999 ▶); data reduction: *SAINT*; program(s) used to solve structure: *SHELXS97* (Sheldrick, 2008 ▶); program(s) used to refine structure: *SHELXL97* (Sheldrick, 2008 ▶); molecular graphics: *SHELXTL* (Sheldrick, 2008 ▶); software used to prepare material for publication: *SHELXTL*.

## Supplementary Material

Crystal structure: contains datablocks global, I. DOI: 10.1107/S1600536811007422/is2677sup1.cif

Structure factors: contains datablocks I. DOI: 10.1107/S1600536811007422/is2677Isup2.hkl

Additional supplementary materials:  crystallographic information; 3D view; checkCIF report

## Figures and Tables

**Table 1:** Hydrogen-bond geometry (Å, °)

*D*—H⋯*A*	*D*—H	H⋯*A*	*D*⋯*A*	*D*—H⋯*A*
C10—H10⋯F1^i^	0.93	2.54	3.147 (2)	123
O2—H2⋯N3^ii^	0.82	2.11	2.819 (2)	145

Symmetry codes: (i) 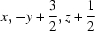; (ii) 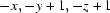.

**Fig. 1.**
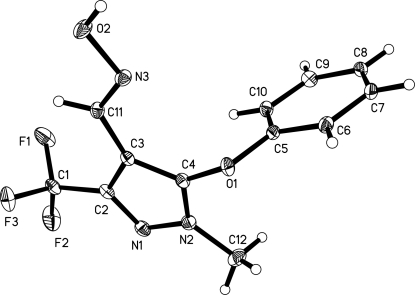
The molecular structure of the title compound, with displacement ellipsoids drawn at the 30% probability level.

**Fig. 2.**
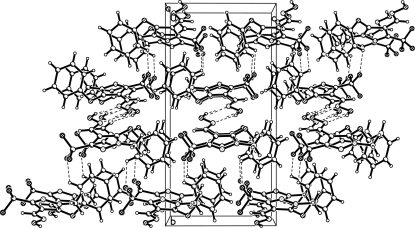
A packing diagram of the title compound viewed along the a axis, with hydrogen bonds drawn as dashed lines.

## supplementary crystallographic information

### Comment

The pyrazole oxime unit plays an important role in many biologically active compounds. A large number of pyrazole oxime derivatives are well acknowledged to possess fungicidal, insecticidal, and acaricidal activities (Hamaguchi *et al.*, 1995). For example, fenpyroximate, a commercial acaricide, has been widely used for the control of mites on many crops (Motoba *et al.*, 1992).

The title compound, (I), is an important intermediate for agrochemicals and drugs. It contains two planes, the pyrazole ring (N2/N1/C2–C4) and the phenyl ring (C5–C10) (Fig. 1). The dihedral angle between the phenyl ring and the pyrazole ring is 96.6 (3)°. In the crystal structure, the molecules are linked by intermolecular C—H···F and O—H···N hydrogen bonds (Table 1 and Fig. 2).

### Experimental

To a stirred solution of hydroxylamine hydrochloride (7.5 mmol) and potassium hydroxide (10 mmol) in ethanol (30 ml), was added 1-methyl-3-(trifluoromethyl)-5-phenoxy-1*H*-pyrazole-4-carbaldehyde (5 mmol) at room temperature. The resulting mixture was heated to reflux for 3 h. The reaction mixture was poured into water (150 ml) and extracted with ethyl acetate (3 × 40 ml). The organic layer was washed with saturated brine (3 × 20 ml), and dried over anhydrous magnesium sulfate. The solvent was evaporated under reduced pressure, then the residue was recrystallized from ethyl acetate to give colourless crystals.

### Refinement

All H atoms were placed in calculated positions, with O—H = 0.82 Å, C—H = 0.93 or 0.96 Å, and included in the final cycles of refinement using a riding model, with *U*_iso_(H) = 1.2*U*_eq_(C) or 1.5*U*_eq_(O, methylC).

### Crystal data

**Table:**

C_12_H_10_F_3_N_3_O_2_	*F*(000) = 584
*M**_r_* = 285.23	*D*_x_ = 1.514 Mg m^−^^3^
Monoclinic, *P*2_1_/*c*	Mo *K*α radiation, λ = 0.71073 Å
Hall symbol: -P 2ybc	Cell parameters from 3687 reflections
*a* = 7.5221 (15) Å	θ = 2.2–27.9°
*b* = 18.282 (4) Å	µ = 0.14 mm^−^^1^
*c* = 9.1002 (18) Å	*T* = 113 K
β = 90.58 (3)°	Monoclinic, colourless
*V* = 1251.4 (4) Å^3^	0.24 × 0.16 × 0.14 mm
*Z* = 4	

### Data collection

**Table:**

Bruker SMART 1000 CCD diffractometer	2185 independent reflections
Radiation source: fine-focus sealed tube	1910 reflections with *I* > 2σ(*I*)
graphite	*R*_int_ = 0.030
φ and ω scans	θ_max_ = 25.0°, θ_min_ = 2.2°
Absorption correction: multi-scan (*SADABS*; Sheldrick, 1996)	*h* = −8→8
*T*_min_ = 0.968, *T*_max_ = 0.981	*k* = −21→21
7050 measured reflections	*l* = −7→10

### Refinement

**Table:**

Refinement on *F*^2^	Primary atom site location: structure-invariant direct methods
Least-squares matrix: full	Secondary atom site location: difference Fourier map
*R*[*F*^2^ > 2σ(*F*^2^)] = 0.037	Hydrogen site location: inferred from neighbouring sites
*wR*(*F*^2^) = 0.101	H-atom parameters constrained
*S* = 1.06	*w* = 1/[σ^2^(*F*_o_^2^) + (0.0599*P*)^2^ + 0.3152*P*] where *P* = (*F*_o_^2^ + 2*F*_c_^2^)/3
2185 reflections	(Δ/σ)_max_ = 0.002
183 parameters	Δρ_max_ = 0.25 e Å^−^^3^
0 restraints	Δρ_min_ = −0.24 e Å^−^^3^

### Special details

**Table:**

Geometry. All e.s.d.'s (except the e.s.d. in the dihedral angle between two l.s. planes) are estimated using the full covariance matrix. The cell e.s.d.'s are taken into account individually in the estimation of e.s.d.'s in distances, angles and torsion angles; correlations between e.s.d.'s in cell parameters are only used when they are defined by crystal symmetry. An approximate (isotropic) treatment of cell e.s.d.'s is used for estimating e.s.d.'s involving l.s. planes.
Refinement. Refinement of *F*^2^ against ALL reflections. The weighted *R*-factor *wR* and goodness of fit *S* are based on *F*^2^, conventional *R*-factors *R* are based on *F*, with *F* set to zero for negative *F*^2^. The threshold expression of *F*^2^ > σ(*F*^2^) is used only for calculating *R*-factors(gt) *etc*. and is not relevant to the choice of reflections for refinement. *R*-factors based on *F*^2^ are statistically about twice as large as those based on *F*, and *R*- factors based on ALL data will be even larger.

### Fractional atomic coordinates and isotropic or equivalent isotropic displacement parameters (Å^2^)

**Table:**

	*x*	*y*	*z*	*U*_iso_*/*U*_eq_	
F1	0.51229 (15)	0.68729 (6)	0.18505 (12)	0.0482 (3)	
F2	0.78938 (15)	0.68491 (7)	0.24060 (12)	0.0473 (3)	
F3	0.65809 (15)	0.58843 (5)	0.16378 (11)	0.0372 (3)	
O1	0.36787 (14)	0.56226 (6)	0.71364 (11)	0.0222 (3)	
O2	0.02826 (15)	0.51556 (7)	0.32441 (12)	0.0296 (3)	
H2	−0.0560	0.4964	0.3661	0.044*	
N1	0.70823 (17)	0.64542 (7)	0.51151 (15)	0.0248 (3)	
N2	0.62765 (17)	0.62190 (7)	0.63485 (14)	0.0228 (3)	
N3	0.15547 (16)	0.53763 (7)	0.42921 (14)	0.0220 (3)	
C1	0.6393 (2)	0.64718 (9)	0.24941 (18)	0.0242 (4)	
C2	0.59492 (19)	0.62818 (8)	0.40404 (17)	0.0203 (3)	
C3	0.43940 (19)	0.59357 (8)	0.45480 (16)	0.0184 (3)	
C4	0.46821 (19)	0.59138 (8)	0.60477 (16)	0.0189 (3)	
C5	0.26932 (19)	0.61152 (8)	0.79966 (16)	0.0190 (3)	
C6	0.1903 (2)	0.58217 (9)	0.92258 (16)	0.0217 (3)	
H6	0.2044	0.5329	0.9455	0.026*	
C7	0.0894 (2)	0.62741 (9)	1.01145 (16)	0.0242 (4)	
H7	0.0346	0.6083	1.0941	0.029*	
C8	0.0696 (2)	0.70110 (9)	0.97772 (17)	0.0237 (4)	
H8	0.0027	0.7314	1.0378	0.028*	
C9	0.1506 (2)	0.72911 (8)	0.85367 (18)	0.0244 (4)	
H9	0.1377	0.7784	0.8309	0.029*	
C10	0.2506 (2)	0.68442 (8)	0.76298 (17)	0.0216 (3)	
H10	0.3040	0.7032	0.6793	0.026*	
C11	0.2899 (2)	0.56673 (8)	0.36830 (16)	0.0201 (3)	
H11	0.2922	0.5709	0.2665	0.024*	
C12	0.7152 (2)	0.62954 (10)	0.77800 (19)	0.0334 (4)	
H12A	0.6683	0.6715	0.8278	0.050*	
H12B	0.8407	0.6356	0.7646	0.050*	
H12C	0.6940	0.5865	0.8356	0.050*	

### Atomic displacement parameters (Å^2^)

**Table:**

	*U*^11^	*U*^22^	*U*^33^	*U*^12^	*U*^13^	*U*^23^
F1	0.0497 (7)	0.0564 (7)	0.0389 (6)	0.0268 (6)	0.0204 (5)	0.0248 (5)
F2	0.0449 (7)	0.0558 (7)	0.0417 (7)	−0.0275 (5)	0.0218 (5)	−0.0041 (5)
F3	0.0598 (7)	0.0258 (5)	0.0262 (5)	0.0001 (5)	0.0137 (5)	−0.0028 (4)
O1	0.0292 (6)	0.0187 (5)	0.0188 (6)	0.0001 (4)	0.0072 (5)	0.0010 (4)
O2	0.0249 (6)	0.0424 (7)	0.0216 (6)	−0.0154 (5)	−0.0038 (5)	0.0028 (5)
N1	0.0202 (7)	0.0250 (7)	0.0294 (7)	−0.0026 (5)	0.0054 (6)	−0.0023 (6)
N2	0.0210 (7)	0.0247 (7)	0.0227 (7)	−0.0007 (5)	0.0001 (5)	−0.0020 (5)
N3	0.0208 (7)	0.0241 (7)	0.0210 (7)	−0.0045 (5)	−0.0018 (5)	0.0003 (5)
C1	0.0220 (8)	0.0214 (8)	0.0293 (8)	−0.0004 (6)	0.0094 (7)	0.0001 (7)
C2	0.0186 (7)	0.0174 (7)	0.0251 (8)	0.0001 (6)	0.0059 (6)	−0.0011 (6)
C3	0.0184 (7)	0.0171 (7)	0.0196 (8)	0.0006 (6)	0.0046 (6)	0.0006 (6)
C4	0.0196 (7)	0.0169 (7)	0.0204 (8)	−0.0005 (6)	0.0033 (6)	0.0001 (6)
C5	0.0187 (7)	0.0222 (8)	0.0162 (7)	−0.0012 (6)	−0.0012 (6)	−0.0027 (6)
C6	0.0238 (8)	0.0228 (8)	0.0186 (8)	−0.0015 (6)	−0.0007 (6)	0.0035 (6)
C7	0.0223 (8)	0.0346 (9)	0.0158 (7)	−0.0019 (7)	0.0016 (6)	0.0022 (7)
C8	0.0204 (8)	0.0313 (9)	0.0194 (8)	0.0002 (6)	0.0008 (6)	−0.0061 (7)
C9	0.0260 (8)	0.0199 (8)	0.0274 (9)	−0.0018 (6)	0.0011 (7)	−0.0007 (6)
C10	0.0239 (8)	0.0227 (8)	0.0183 (8)	−0.0033 (6)	0.0026 (6)	0.0021 (6)
C11	0.0226 (8)	0.0211 (7)	0.0167 (7)	−0.0022 (6)	0.0028 (6)	0.0016 (6)
C12	0.0325 (9)	0.0381 (10)	0.0295 (9)	−0.0027 (8)	−0.0106 (7)	−0.0037 (8)

### Geometric parameters (Å, °)

**Table:**

F1—C1	1.3350 (19)	C5—C6	1.381 (2)
F2—C1	1.3260 (19)	C5—C10	1.381 (2)
F3—C1	1.3354 (19)	C6—C7	1.388 (2)
O1—C4	1.3601 (18)	C6—H6	0.9300
O1—C5	1.4089 (18)	C7—C8	1.389 (2)
O2—N3	1.4034 (17)	C7—H7	0.9300
O2—H2	0.8200	C8—C9	1.386 (2)
N1—C2	1.329 (2)	C8—H8	0.9300
N1—N2	1.3512 (19)	C9—C10	1.388 (2)
N2—C4	1.348 (2)	C9—H9	0.9300
N2—C12	1.460 (2)	C10—H10	0.9300
N3—C11	1.274 (2)	C11—H11	0.9300
C1—C2	1.491 (2)	C12—H12A	0.9600
C2—C3	1.412 (2)	C12—H12B	0.9600
C3—C4	1.380 (2)	C12—H12C	0.9600
C3—C11	1.452 (2)		
			
C4—O1—C5	116.97 (11)	C5—C6—C7	118.88 (14)
N3—O2—H2	109.5	C5—C6—H6	120.6
C2—N1—N2	104.25 (12)	C7—C6—H6	120.6
C4—N2—N1	111.62 (13)	C6—C7—C8	120.46 (14)
C4—N2—C12	127.78 (14)	C6—C7—H7	119.8
N1—N2—C12	120.58 (13)	C8—C7—H7	119.8
C11—N3—O2	111.28 (12)	C9—C8—C7	119.43 (15)
F2—C1—F1	107.08 (14)	C9—C8—H8	120.3
F2—C1—F3	106.74 (13)	C7—C8—H8	120.3
F1—C1—F3	105.38 (14)	C8—C9—C10	120.79 (15)
F2—C1—C2	112.16 (14)	C8—C9—H9	119.6
F1—C1—C2	112.09 (13)	C10—C9—H9	119.6
F3—C1—C2	112.92 (13)	C5—C10—C9	118.60 (14)
N1—C2—C3	113.14 (14)	C5—C10—H10	120.7
N1—C2—C1	119.44 (13)	C9—C10—H10	120.7
C3—C2—C1	127.41 (14)	N3—C11—C3	121.26 (14)
C4—C3—C2	102.36 (13)	N3—C11—H11	119.4
C4—C3—C11	129.75 (14)	C3—C11—H11	119.4
C2—C3—C11	127.89 (14)	N2—C12—H12A	109.5
N2—C4—O1	120.87 (13)	N2—C12—H12B	109.5
N2—C4—C3	108.62 (13)	H12A—C12—H12B	109.5
O1—C4—C3	130.46 (13)	N2—C12—H12C	109.5
C6—C5—C10	121.83 (14)	H12A—C12—H12C	109.5
C6—C5—O1	115.79 (13)	H12B—C12—H12C	109.5
C10—C5—O1	122.38 (13)		
			
C2—N1—N2—C4	−0.42 (16)	C5—O1—C4—C3	−103.49 (18)
C2—N1—N2—C12	178.30 (14)	C2—C3—C4—N2	−0.38 (16)
N2—N1—C2—C3	0.17 (17)	C11—C3—C4—N2	178.89 (15)
N2—N1—C2—C1	179.17 (13)	C2—C3—C4—O1	−177.58 (14)
F2—C1—C2—N1	−4.1 (2)	C11—C3—C4—O1	1.7 (3)
F1—C1—C2—N1	−124.64 (15)	C4—O1—C5—C6	−170.60 (13)
F3—C1—C2—N1	116.52 (16)	C4—O1—C5—C10	10.2 (2)
F2—C1—C2—C3	174.70 (14)	C10—C5—C6—C7	0.0 (2)
F1—C1—C2—C3	54.2 (2)	O1—C5—C6—C7	−179.25 (12)
F3—C1—C2—C3	−64.6 (2)	C5—C6—C7—C8	−0.5 (2)
N1—C2—C3—C4	0.13 (17)	C6—C7—C8—C9	0.5 (2)
C1—C2—C3—C4	−178.78 (15)	C7—C8—C9—C10	0.1 (2)
N1—C2—C3—C11	−179.16 (14)	C6—C5—C10—C9	0.6 (2)
C1—C2—C3—C11	1.9 (3)	O1—C5—C10—C9	179.76 (13)
N1—N2—C4—O1	178.05 (12)	C8—C9—C10—C5	−0.6 (2)
C12—N2—C4—O1	−0.6 (2)	O2—N3—C11—C3	−179.74 (13)
N1—N2—C4—C3	0.52 (17)	C4—C3—C11—N3	2.3 (3)
C12—N2—C4—C3	−178.08 (15)	C2—C3—C11—N3	−178.62 (15)
C5—O1—C4—N2	79.59 (17)		

### Hydrogen-bond geometry (Å, °)

**Table:**

*D*—H···*A*	*D*—H	H···*A*	*D*···*A*	*D*—H···*A*
C10—H10···F1^i^	0.93	2.54	3.147 (2)	123
O2—H2···N3^ii^	0.82	2.11	2.819 (2)	145

Symmetry codes: (i) *x*, −*y*+3/2, *z*+1/2; (ii) −*x*, −*y*+1, −*z*+1.
